# Concomitant Nephrotic Syndrome and Cryoglobulinemia in a Case of Malignant Mesothelioma

**DOI:** 10.1155/2022/8677293

**Published:** 2022-09-28

**Authors:** Kei Nagai, Hiroaki Tachi, Kohei Inoue, Atsushi Ueda

**Affiliations:** ^1^Department of Nephrology, Hitachi General Hospital, 2-1-1 Jonan-cho, Hitachi, Ibaraki 317-0077, Japan; ^2^Department of Nephrology, Faculty of Medicine, University of Tsukuba, 1-1-1 Tennodai, Tsukuba, Ibaraki 305-8575, Japan; ^3^Department of Respiratory Medicine, Hitachi General Hospital, 2-1-1 Jonan-cho, Hitachi, Ibaraki 317-0077, Japan

## Abstract

Malignant pleural mesothelioma is rarely associated with nephrotic syndrome. Cryoglobulinemia is found in various pathological statuses, such as hepatitis C virus infection but rarely in malignant neoplasms. We recently encountered a patient with malignant mesothelioma coincident with nephrotic syndrome and cryoglobulinemia in the course of chemotherapy. A 60-year-old man employed as a building painter was diagnosed with malignant mesothelioma by lung biopsy two years earlier and was started on chemotherapy. Nivolumab seemed effective in controlling mesothelioma, but skin immune-related adverse events occurred during the course of treatment. After discontinuation of nivolumab and administration of gemcitabine as an alternative therapy, the patient was referred to a nephrologist because of the subsequent development of edema, renal injury, and proteinuria. Following the investigation, he was diagnosed with nephrotic syndrome and cryoglobulinemia with C4-dominant cold activation. However, a percutaneous renal biopsy could not be performed due to persistent severe cough induced by pleural involvement. The patient died a little over three years after the pathological diagnosis of pleural mesothelioma. Our case had three key features nephrotic syndrome was possibly associated with malignant mesothelioma; cryoglobulinemia occurred in malignant mesothelioma; and concomitant nephrotic syndrome and cryoglobulinemia occurred after chemotherapy. Unfortunately, our rare case lacks a basis in renal pathology or evidence of links between the pathogenesis of malignant mesothelioma, cryoglobulinemia, and nephrotic syndrome. This case does not provide a causal mechanism, but may be worth adding to the case list as one of the rare renal involvement in a patient with malignant mesothelioma.

## 1. Introduction

Nephrotic syndrome is rarely associated with malignant pleural mesothelioma but is well-known to be induced as a paraneoplastic syndrome [1]. Although not leading to any cohort studies or case series, sporadic cases of nephrotic syndrome associated with malignant mesothelioma have been reported [2–16]. While most cases are assumed to represent nephrotic syndrome secondary to cancer, nephrotic syndrome may develop during the treatment of mesothelioma, and a mechanism specifically related to chemotherapy has also been postulated [15].

Cryoglobulinemia is found in various pathologic statuses. Before the appearance of specific antiviral treatments, the main etiology was chronic hepatitis C virus (HCV) infection, and currently, cryoglobulinemia is mainly associated with systemic autoimmune diseases, malignant neoplasms, and cases without an identified etiology (i.e., essential cryoglobulinemia) [17, 18]. Malignant mesothelioma with the development of cryoglobulinemia is very rare but has been reported in some cases [19].

In addition to such rare cases, we recently encountered a patient with malignant mesothelioma with concomitant nephrotic syndrome and cryoglobulinemia during the course of chemotherapy.

## 2. Case Presentation

**Figure 1:**
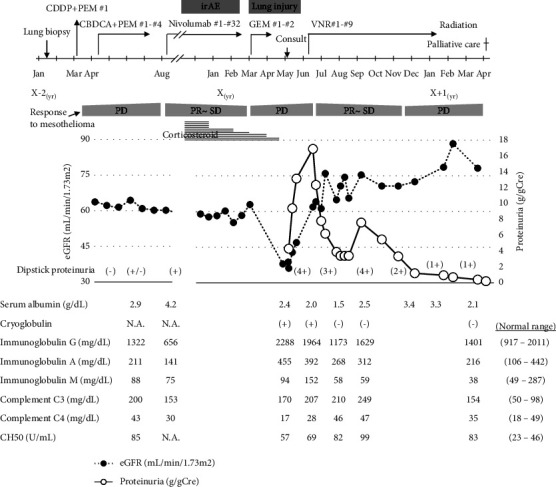
The clinical course of the patient. Two years earlier, the patient had been diagnosed with malignant mesothelioma by lung biopsy and treated with chemotherapy. Cisplatin plus pemetrexed (CDDP + PEM) and carboplatin plus pemetrexed (CBDCA + PEM) were determined to result in an insufficient response, and nivolumab was considered to have induced immune-related adverse events and was discontinued. After starting the administration of gemcitabine (GEM), drug-induced lung injury occurred. He was also referred to a nephrologist because of the subsequent development of edema, renal injury, and proteinuria. After investigations and careful consideration, vinorelbine (VNR) was administered to treat the nephrotic syndrome with cryoglobulinemia due to the progression of malignant mesothelioma. Response of chemotherapy to malignant mesothelioma is presented as partial response (PR), stable disease (SD), and progressive disease (PD). Palliative care was subsequently provided, along with radiation therapy for metastatic bone lesions and antibiotics against bacterial pneumonia. The patient died a little over 3 years after the pathological diagnosis of mesothelioma without recurrence of nephrotic syndrome in the end-stage of cancer.

A 60-year-old man visited a nephrologist due to edema, renal injury, and proteinuria. The man was a building painter and had developed massive lateral pleural effusions three years earlier. He was finally diagnosed with malignant mesothelioma from a thoracoscopic lung biopsy 2 years earlier and was started on chemotherapy (Figure 1). Cisplatin plus pemetrexed (CDDP + PEM) was administered as first-line therapy, followed by carboplatin plus pemetrexed (CBDCA + PEM) as second-line treatment. This regimen proved ineffective and the disease progressed, so the patient was switched to nivolumab. Nivolumab seemed effective in controlling mesothelioma, but skin immune-related adverse events (irAEs) occurred during the course of treatment. Prednisolone (20 mg/day) was given to alleviate irAEs and was gradually tapered. After discontinuation of nivolumab and administration of gemcitabine (GEM) as fourth-line therapy, drug-induced lung injury occurred and GEM was also discontinued. The patient was referred to a nephrologist after edema, renal injury and proteinuria developed.

At the first visit, as shown in Table 1, laboratory data showed massive proteinuria (4.3 g/day) and hypoalbuminemia (2.4 g/day), suggesting nephrotic syndrome. Notable findings were newly developed polyclonal gammopathy (IgG 2,288 mg/dL, IgA 455 mg/dL, and IgM 94 mg/dL) and characteristic low complementemia (C3 170 mg/dL, C4 17 mg/dL, and CH50 57 U/mL), suggesting C4-dominant cold activation. Cryoglobulins were qualitatively found to be positive. Other causes of secondary cryoglobulinemia, such as hepatitis B virus and HCV chronic infections were serologically excluded. Since cryoglobulin might occur with antigens from malignant disease, the patient was administered another line of chemotherapy for nephrotic syndrome with cryoglobulinemia due to the progression of malignant mesothelioma after careful consideration and informed consent. Nephrotic syndrome gradually improved along with the administration of vinorelbine (VNR) as the fifth-line treatment (Figure 1). Low serum C4, cryoglobulinemia, and nephrotic range of proteinuria had disappeared after VNR therapy. Percutaneous renal biopsy could not be performed due to persistent severe cough induced by pleural involvement. The clinical diagnosis was thought to be nephrotic syndrome with cryoglobulinemia due to the progression of malignant mesothelioma. Thereafter, palliative care was provided, along with radiotherapy for metastatic bone lesions and antibiotics against bacterial pneumonia. The patient died a little over three years after pathological diagnosis of mesothelioma without recurrence of nephrotic syndrome in the end-stage of cancer.

## 3. Discussion

The present case showed three features newly developed nephrotic syndrome was possibly associated with malignant mesothelioma; cryoglobulinemia occurred in malignant mesothelioma; and concomitant transient nephrotic syndrome and cryoglobulinemia occurred after the use of nivolumab or GEM, but not VNR. Unfortunately, this case lacked a basis in renal pathology or evidence of links between the pathogenesis of malignant mesothelioma, cryoglobulinemia, and nephrotic syndrome. However, we discuss here the causal relationship between the three events based on the literature as much as possible.

We reviewed 15 publications presenting cases of nephrotic syndrome with malignant mesothelioma at various sites [2–16]. Seven cases included information on complement levels, all of which were within the normal range. A description of cryoglobulins was absent in all previous cases (Table 2). Most of the renal pathology involved minimal-change disease in patients with malignant mesothelioma and nephrotic syndrome [12], accounting for 9 of the 15 cases (60%) in Table 2. Three cases of membranous nephropathy were identified, followed by one case each of focal segmental glomerulosclerosis and mesangial proliferative nephritis, but no reports of membranous proliferative glomerulonephritis, which may occur in cryoglobulinemia. Meanwhile, definitively assessing the pathological characteristics of this case is difficult because renal biopsy could not be performed at the moment of nephrotic syndrome due to the condition of the patient. Nevertheless, the result of the high selectivity of proteinuria (index, 0.09; Table 1) suggests the possibility of minimal-change disease, consistent with previous cases.

Cryoglobulinemia-associated glomerulonephritis is most commonly seen in the context of hepatitis C infection and occasionally seen in relation to lymphoma and malignant neoplasm [18, 20–22]. In contrast with renal disease associated with HCV, renal involvement in patients with cryoglobulinemia not associated with HCV has only been poorly described, and few cases have been reported [23]. In an analyzed series of 20 cases without HCV-associated cryoglobulinemia, renal involvement was characterized by nephrotic range proteinuria in 75% of patients and renal failure in 85% of patients (mean glomerular filtration rate, 46 mL/min/1.73 m^2^) [23]. Membranoproliferative glomerulonephritis with subendothelial deposits was observed in all kidney specimens [23]. Generally, therapeutic options for the disease include removal of circulating cryoglobulin by plasmapheresis, immunosuppression, and pharmacological treatment to address the underlying cause of protein production. After excluding the possibility of any infectious diseases, including viral hepatitis, and careful consideration, we tried suppression of cancer progression by next-line chemotherapy with VNR after the diagnosis of nephrotic syndrome. In this case, cryoglobulin disappeared without apheresis or immunosuppressive drugs, suggesting that one of the relevant mechanisms was anticancer therapy reducing the tumor burden as an antigen against cryoglobulin.

Another possibility for the pathogenesis was that the concomitant nephrotic syndrome and cryoglobulinemia were driven by chemotherapy. In terms of the time course, in this case, the suspect drug was nivolumab or GEM, but not VNR. Only one study suggested that anti-PD-1-related cryoglobulinemia during nivolumab treatment in a patient with lung cancer [24]. He was treated with prednisone and cryoprecipitates disappeared after 26 days of steroid therapy. The patient continued to receive nivolumab for a total of eight cycles with no recurrence of cryoglobulinemia [24]. Nivolumab is also known as a rare cause of nephrotic syndrome during cancer therapy, represented by minimal-change disease [25], membranous nephropathy [26], membranoproliferative glomerulonephritis [27], and renal vasculitis [28]. A hemolytic uremic syndrome is the most perilous adverse effect of gemcitabine, with an incidence of approximately 0.15% based on reported cases [29]. GEM-induced secondary thrombotic microangiopathy may be diagnosed as a nephrotic syndrome [30]. Membranous nephropathy as a cause of nephrotic syndrome was also found in a case treated using GEM [31]. Collectively, both nivolumab and GEM could cause nephrotic syndrome, while no previous reports have shown any association between GEM and cryoglobulinemia. Therefore, if we consider nephrotic syndrome and cryoglobulinemia to represent linked complications rather than coincidences, the suspect drug is most likely nivolumab, not GEM.

To the best of our knowledge, this represents the first description of concomitant nephrotic syndrome and cryoglobulinemia in a case of malignant mesothelioma. Although no causal mechanisms or pathological findings were identified, this case may be worth adding to the case list as a rare coincidence in a patient with malignant mesothelioma.

## Figures and Tables

**Table 1:** Laboratory findings.

Urinalysis		Blood chemistry tests (cont.)	
Gravity	1.009	Sodium	136 mmol/L
Protein	3+	Chloride	104 mmol/L
Sugar	Negative	Potassium	3.0 mmol/L
Blood	1+	Corrected calcium	10.0 mg/dL
Sediment		Phosphate	3.0 mg/dL
Red blood cells	5–9/HPF	Total bilirubin	0.3 mg/dL
White blood cells	10–19/HPF	Aspartate aminotransferase	22 U/L
Urinary biochemical tests	Alanine aminotransferase	26 U/L	
Daily urinary protein	4.3 g/24 hr	Lactate dehydrogenase	292 U/L
Selectivity index	0.09	Alkaline phosphatase	167 U/L
Bence-Jones protein	Negative	Creatine kinase	73 U/L
Complete blood count	Total cholesterol	197 mg/dL	
White blood cells	10600/mL	LDL cholesterol	129 mg/dL
Neutrophils	58%	Triglyceride	191 mg/dL
Eosinophils	12%	Glucose	103 mg/dL
Basophils	3%	Hemoglobin A1c	5.1%
Lymphocytes	15%	Serology	
Monocytes	11%	C-reactive protein	3.38 mg/dL
Hemoglobin	11.0 g/dL	HBs antigen	Negative
Platelets	57.3 × 104/mL	Anti-HCV antibody	Negative
Coagulation tests	Immunoglobulin G	2288 mg/dL	
PT-INR	1.13	Immunoglobulin A	455 mg/dL
APTT	28.5 sec	Immunoglobulin M	94 mg/dL
APTT, ctrl	33.5 sec	Complement 3	170 mg/dL
Fibrinogen	760 mg/dL	Complement 4	17 mg/dL
von Willebrand factor	341% (60–170%)	CH50	57.0 U/mL
Blood chemistry tests	Rheumatoid factor	33 IU/mL	
Total protein	7.2 g/dL	Antinuclear antibody	x40 >
Albumin	2.4 g/dL	Anti-dsDNA-Ab	< 10 U/mL
Uric acid	10.1 mg/dL	PR3-ANCA	< 1.0 U/mL
Urea nitrogen	14.2 mg/dL	MPO-ANCA	< 1.0 U/mL
Creatinine	1.59 mg/dL	Cryoglobulin	(+)

HPF: high-power field; PT-INR: prothrombin time-international normalized ratio; APTT: activated partial thromboplastin time; CH50:50% hemolytic unit of complement; LDL: low-density lipoprotein; HBs: hepatitis B surface; HCV: hepatitis C virus; dsDNA-Ab: double-strand deoxyribonucleic acid antibody; PR3-ANCA: proteinase-3-antineutrophil cytoplasmic antibodies; MPO-ANCA: myeloperoxidase-antineutrophil cytoplasmic antibodies.

**Table 2:** The literature review of cryoglobulin and renal diseases in malignant mesothelioma.

First Author	Year	Disease site	Complement	Cryoglobulin	Chemotherapy for mesothelioma	Renal Pathology
Schroeter	1986	Pleura	Normal	NA	Cyclophosphamide, doxorubicin, hydrochloride, and decarbonize	MCNS
Venzano	1990	Pleura	NA	NA	NA	MCNS
Absy	1992	Pleura	NA	NA	Carboplatin	FSGS
Tanaka	1994	Pleura	Normal	NA	Carboplatin, etoposide	MesPGN
Galesic	2000	Pleura	Normal	NA	None (corticosteroid)	MN
Sakamoto	2000	Pleura	NA	NA	None (surgery)	MN
Farmer	2001	Pleura and brain metastasis	NA	NA	None (corticosteroid)	MCNS
Bacchetta	2009	Testis	Normal	NA	Cisplatin and pemetrexed	MCNS
Li	2010	Pleura	Normal	NA	Gemcitabine and carboplatin	MCNS
Dogan	2012	Pleura	NA	NA	None (surgery)	Not examined
Suzuki	2014	Pleura	Normal	NA	None (conservative)	MCNS
Tsukamoto	2015	Pleura, pulmonary, and skull metastasis	NA	NA	Carboplatin, pemetrexed, and gemcitabine	MCNS
Pu	2016	Pleura	NA	NA	Cisplatin and pemetrexed	MN
Bickel	2016	Pleura	NA	NA	Carboplatin, pemetrexed, vinorelbine, and pembrolizumab	MCNS
Yildiz	2016	Pleura	Normal	NA	None (corticosteroid)	MCNS
Nagai	Present case	Pleural mesothelioma and bone metastasis	Low C4	Positive	Cisplatin, pemetrexed, carboplatin, nivolumab, gemcitabine, and vinorelbine	Not examined

MCNS: minimal-change nephrotic syndrome; FSGS: focal segmental glomerulosclerosis; MesPGN: mesangial proliferative glomerulonephritis: MN: membranous nephropathy.

## Data Availability

The data generated during the current case are available from the corresponding author upon reasonable request.
